# GABA and Glx levels in cortico-subcortical networks predict catecholaminergic effects on response inhibition

**DOI:** 10.1177/02698811251340893

**Published:** 2025-06-20

**Authors:** Anna Helin Koyun, Annett Werner, Paul Kuntke, Veit Roessner, Christian Beste, Ann-Kathrin Stock

**Affiliations:** 1 Cognitive Neurophysiology, Department of Child and Adolescent Psychiatry, Faculty of Medicine, TU Dresden, Germany; 2 Institute of Diagnostic and Interventional Neuroradiology, TU Dresden, Germany; 3 German Center for Child and Adolescent Health (DZKJ), Partner Site Leipzig/Dresden, Dresden, Germany

**Keywords:** ^1^H-MRS, GABA, Glx, dopamine and norepinephrine, response inhibition

## Abstract

**Background:**

Cortico-subcortical networks play a fundamental role in cognitive control. Within these circuits, neurotransmitters such as gamma-aminobutyric acid (GABA), glutamate, and catecholamines crucially modulate response control and (motor) response inhibition. Despite the evident interrelation between these transmitter systems, the role of baseline GABA and glutamate-glutamine (Glx) levels in predicting/influencing catecholaminergic effects has remained rather unclear.

**Aims:**

Addressing this knowledge gap, we investigated the question how much (and which facets) of behavioral effects attributed to catecholamines are due to GABAergic and glutamatergic levels in control-relevant cortical networks.

**Methods:**

Using proton-magnetic resonance spectroscopy, we assessed baseline GABA+ and Glx levels within the striatum, the anterior cingulate cortex, and the (pre-)supplementary motor cortex ((pre-)SMA), and their predictive value for catecholaminergic modulation of response selection and inhibition performance. For this purpose, we administered low and high doses of methylphenidate (MPH) to healthy adults and examined whether baseline GABA+ and Glx were associated with dose-dependent MPH effects on response control.

**Results/Outcomes:**

For the first time in a sample of healthy adults, we demonstrate that GABA+/Glx levels in cognitive control-relevant cortical areas are indicative of the magnitude of MPH-induced effects on response inhibition. Specifically, striatal GABA+/Glx levels predicted better response inhibition performance under the administration of low MPH doses. In contrast, (pre-)SMA GABA+/Glx levels were associated with high MPH dose-induced impairments of response inhibition performance.

**Conclusion/Interpretation:**

The predictive relevance of GABA+/Glx levels for MPH dose-dependent effects on cognitive control processes provides valuable insights into the neural mechanisms underlying the previously reported heterogeneous MPH effects.

## Introduction

Response inhibition is a fundamental cognitive process that is crucial for maintaining effective behavioral control (for review, see Bari and Robbins, 2013; Diamond, 2013). The successful implementation of this faculty varies among individuals, and dysfunctions are frequently associated with various neuropsychiatric disorders. On a functional neuroanatomical level, cortico-subcortical networks are integral to (motor) response control and inhibition (Bari and Robbins, 2013), but the specific role of amino acid neurotransmitters in these structures has remained less understood. This knowledge is however vital for developing targeted interventions and advancing our knowledge of cognitive processes that contribute to goal-directed behavior.

The pre-supplementary and supplementary motor areas (pre-SMA, SMA; Bari and Robbins, 2013), the anterior cingulate cortex (ACC; Adams et al., 2017), and the inferior frontal cortex (Aron et al., 2004) have been postulated to be part of a response inhibition network. Both the (pre-)SMA and ACC modulate automatic motor activation and the suppression of prepotent or inappropriate responses in favor of more adaptive behaviors (Rushworth et al., 2007; Shenhav et al., 2018; Wolpe et al., 2022). Specifically, the ACC is crucially involved in behavioral flexibility and response selection, while the (pre-)SMA is involved in monitoring (motor) conflicts and motor response inhibition. The inferior frontal cortex is interconnected with the basal ganglia. Microstructural properties of this latter region and inhibitory neurotransmitters (especially in the striatum) have been shown to facilitate (motor) response selection/inhibition and inhibit the activation of interfering actions (Beste et al., 2014; Redgrave et al., 2011).

Inherent neurobiochemical properties of these control-relevant cortical areas play key roles in orchestrating the complex and dynamic processes defining cognitive control. Gamma-aminobutyric acid (GABA) is a critical modulator of neuronal activity, primarily by exerting inhibitory effects, especially on interneurons in the striatum and cingulate areas (Adams et al., 2017; Beste et al., 2014: 200; Quetscher et al., 2015). In healthy adults, GABAergic levels in the striatum and ACC play a key role for fine-tuned response control and motor response inhibition (Quetscher et al., 2015; Silveri et al., 2013; Yildiz et al., 2014). Conversely, the glutamatergic system drives the excitatory part of neurotransmission, with glutamate receptors influencing synaptic plasticity and transmission, thereby modulating the function of fronto-striatal circuits (Cheng et al., 2017). Evidence suggests an important role of glutamatergic projections from the ACC and SMA onto striatal neurons for cognitive/response control and inhibition (Li et al., 2020; Naaijen et al., 2018; Wolff et al., 2019). Glutamatergic inputs from executive cortical networks provide excitatory inputs to SMA neurons, which is crucial for the planning, rapid initiation, and execution of motor responses as well as sensorimotor integration. In healthy adults, glutamate concentrations in the (pre-)SMA and striatum are associated with successful regulation of inhibitory control (Weerasekera et al., 2020). Overall, the complex interplay between GABAergic inhibition and glutamatergic excitation (particularly within the striatum, SMA, and ACC) dynamically contributes to the complex mechanisms of response control and inhibition, enabling quick movement adjustments in alignment with behavioral goals.

Notably, the sensitivity of glutamatergic and GABAergic synapses is affected by other neurotransmitter systems, particularly catecholamines, which are intricately involved in regulating the response inhibition network and cognitive control mechanisms (Manz et al., 2021; Plenz, 2003; Tritsch and Sabatini, 2012). Theoretical and empirical evidence shows that catecholamine levels follow an inverted U-shaped function with either too low or too high levels being suboptimal for performance (Cools and D’Esposito, 2011; Robbins and Arnsten, 2009). Dopaminergic pathways innervating cortical and striatal structures regulate the delicate catecholamine balance required for optimal cognitive functioning by fine-tuning the sensitivity of GABAergic and glutamatergic synapses (Beaulieu and Gainetdinov, 2011; Carlsson et al., 1999; Maier et al., 2020; Vallone et al., 2000; Verger et al., 2020). It is, therefore, of high relevance to investigate the dynamic interplay between baseline amino acid transmitters and catecholamines within the striatum, ACC and SMA, and their predictive influence on cognitive control processes. The results will enhance our understanding of the degree to which GABAergic and glutamatergic signaling in control-relevant cortical networks modulate catecholaminergic effects on behavior.

One way to modulate the catecholaminergic system is by administering methylphenidate (MPH). MPH acts as a mixed dopamine (DA) and norepinephrine (NE) reuptake inhibitor blocking transporters, thereby increasing DA and NE efflux/extracellular levels in the prefrontal cortex and striatum (Bymaster, 2002; Dipasquale et al., 2020; Spencer et al., 2012). MPH-induced catecholaminergic enhancement decreases glutamate levels in preforntal cortex (PFC) and striatal areas in humans (Carrey et al., 2002), and targets glutamate receptors in PFC neurons in a dose-dependent way (Cheng et al., 2014; Urban et al., 2013). Besides, animal studies reported the obstruction of GABAergic transmission by activation of dopamine D4 receptor as a result of single-dose MPH administration (Erlij et al., 2012). Acute MPH-induced increases in catecholaminergic signaling have been associated with an improved ability to distinguish between relevant and irrelevant information (Aston-Jones and Cohen, 2005; Mückschel et al., 2017). Other accounts however report performance impairments after single-dose MPH administrations (Bensmann et al., 2019; Linssen et al., 2014; Mückschel et al., 2020a), further stressing that MPH effects may differ across doses and situations.

We investigated neural substrates contributing to the MPH effects using edited proton-magnetic resonance spectroscopy (^1^H-MRS) to measure baseline GABA+ (GABA and macro molecules) and Glx (glutamate and glutamine) concentrations in the striatum, SMA, and ACC of healthy adults, and relate those to the magnitude and direction of MPH-induced effects. To examine behavioral differences in cognitive/response control, a combination of a Simon task and a Go/NoGo task (Chmielewski and Beste, 2017; Wendiggensen et al., 2022) was used, allowing to investigate interference effects during both response inhibition and execution. In this regard, it is crucial to consider that the interrelation between cognitive performance and catecholamine levels often reflects an inverted U-shaped function (Cools and D’Esposito, 2011; Robbins and Arnsten, 2009). Consequently, interindividual differences in baseline transmitter levels likely influence performance and the effort needed to perform well (Colzato et al., 2016; Takei et al., 2016), and mark different starting points/distances until optimal performance is reached. Considering the close interrelation of catecholamines and amino acid transmitters, we expect baseline levels of GABA+ and Glx in cortical regions relevant for cognitive control (i.e., striatum, SMA, and ACC) to shift the amount of catecholamines needed to induce an “optimal” performance in the task at hand. Different degrees of catecholaminergic stimulation (operationalized by different MPH doses) may therefore push individuals to either optimal or suboptimal performance. We expect low MPH doses to result in better response control, as compared to high MPH doses, because the latter likely pushes individuals beyond an optimal point. We hypothesize that interindividual differences in amino acid transmitter levels in the SMA, striatum, and ACC relate to the size and direction of MPH effects. GABA+ and Glx were assumed to differentially predict possible dose-dependent effects on the behavioral level in a brain region-specific manner. Based on currently available data (Maier et al., 2020; Solleveld et al., 2017), baseline GABA+ and Glx ratios in the striatum and SMA were expected to be predictive of MPH-induced effects on response control. Furthermore, striatal GABA+ was deemed more relevant than Glx levels in explaining behavioral/MPH effect variance due to the predominance of GABAergic medium spiny neurons (MSNs) in the striatum (Bolam et al., 2000). In the SMA, both GABA+ and Glx levels were expected to hold significance in predicting MPH-induced effects, as there is a relative equilibrium between these neurotransmitters. Conversely, we hypothesized that GABA+, but not Glx levels, in the ACC explain variance in MPH effects (Maier et al., 2020).

## Methods and materials

### Ethical approval

Participants provided written informed consent before the experiment and received financial compensation upon completion. The study was approved by the ethics committee of TU Dresden (project number: EK 420092015) and conducted in accordance with the declaration of Helsinki.

### Participants

*N* = 78 young and healthy adults participated in this study. All participants were between 20 and 31 years old, with normal or corrected-to-normal vision, no current/reported history of psychiatric, neurologic, or developmental disorders, and did not use CNS-affecting medication. After initial data inspection, *n* = 14 participants were excluded from the subsequent analyses (for details, see Supplemental Material). This resulted in a final sample of *n* = 64 (mean age 25.30 ± 0.396 SEM; 28 females) participants. Specifically, *n* = 31 participants (24.87 ± 0.546 years; 15 females) were in the low MPH dose group, and *n* = 33 (25.70 ± 0.570 years; 13 females) in the high MPH dose group.

### Experimental design

A double-blind MPH/placebo crossover design was used. Participants underwent one baseline MRS measurement (typically right before the first appointment on the same day) and two experimental sessions spaced 7 days apart. Pseudorandomization, as defined by the between-subject factors of MPH dose (low vs. high) and order of drug administration (MPH on the first vs. second appointment), resulted in a balanced gender ratio within and between each subgroup. Other than that, the group assignment was completely random.

In a double-blind fashion, participants received the respective MPH dose (0.25 or 0.75 mg/kg body weight) on one of the appointments and an identical-looking placebo (lactose) on the other. On both appointments, the experimental task started approximately 120 min after drug intake, as MPH plasma levels (i.e., highest drug concentration in plasma) peak around 1–3 h, while maximum behavioral effects occur about 2 h after oral administration (Challman and Lipsky, 2000; Kimko et al., 1999; Wolraich and Doffing, 2004).

### MRS acquisition and voxel placement

**Figure 1.**
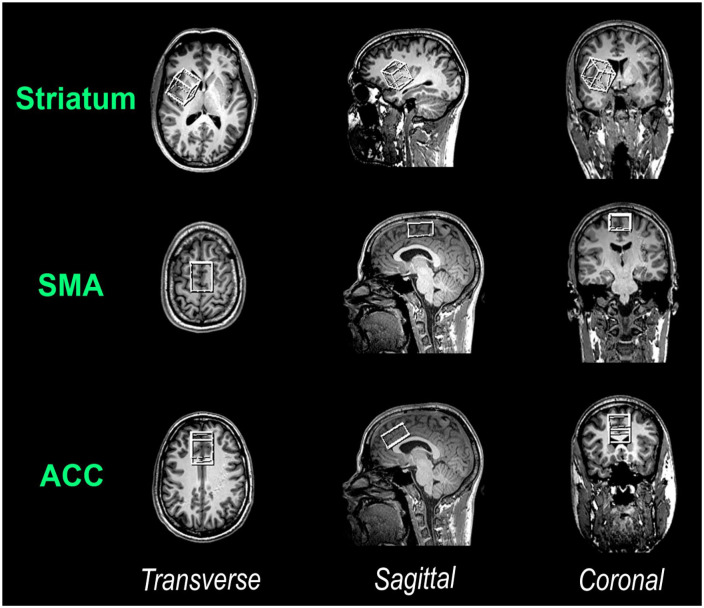
Illustration of the VOI placements. Depicted are the VOIs (from top to bottom): in the right striatum, the (pre-)SMA and ACC (note that sides are mirrored in this scanner output). ACC: anterior cingulate cortex; SMA: supplementary motor cortex; VOI: voxel of interest

MRI and MRS data were acquired with a Siemens 3T Prisma scanner (Siemens Healthineers, Erlangen, Germany) using a 32-channel (receive-only) head rf coil. Initially, high-resolution structural images in sagittal orientation were obtained using a 3D T1-weighted Magnetization Prepared Rapid Gradient Echo sequence (1 mm isovoxel, TE/TR/TI: 2.29 ms/2.3s/0.9 s). Subsequently, these structural images were reconstructed for precise voxel placements. In the current study, MRS was used to quantify brain metabolite (GABA+, Glx, and N-acetylaspartate) concentrations in the striatum, SMA and ACC. Separate voxels of interest (VOIs) were individually positioned for each of these cortical regions. Figure 1 illustrates the VOI placements. Specifically, a 30 × 30 × 30 mm VOI was placed in the right striatum, and a 20 × 30 × 40 mm VOI was positioned in a way that left and right (pre-)SMA were covered. Additionally, a 20 × 30 × 40 mm VOI was placed over the midline to cover large parts of both the left and right ACC (including only relatively small fractions of neighboring brain regions).

To further optimize spectral resolution, manual shimming was performed for each of the VOIs in addition to the inbuilt shim routine. The shimming criterion was a full-width at half-maximum (FWHM) value below 20 Hz for the unsuppressed water signal. To obtain GABA+ and Glx values, we then ran the Center for Magnetic Resonance Research MEGA-PRESS (Mescher-Garwood point-resolved spectroscopy) sequence (echo time TE/repetition time TR = 68/3000 ms, edit ON acquisitions = 128, edit OFF acquisitions = 128) developed by Edward J. Auerbach and Małgorzata Marjańska and provided by the University of Minnesota (Govindaraju et al., 2000; Marjańska et al., 2013; Tremblay et al., 2014) based on a C2P license agreement with Siemens Healthineers AG Germany. The obtained spectra (“edit on”/“edit off”) were exported as raw data (*.rda) from the Siemens Spectroscopy subroutine. After calculation of the difference spectra (“edit on”—“edit off”), GABA+, Glx, and total N-acetylaspartate (tNAA; NAA + N-acetylaspartylglutamate) were quantified as ratios using LCModel software (v6.3-1H, copyright Stephen Provencher, Canada). Representative examples of MEGA-PRESS-edited spectra with LCModel fitting of all VOIs are provided in the Supplemental Material. Basis sets for MEGA-PRESS were delivered by Ulrike Dydak’s MRS Lab at Purdue University (West Lafayette, Indiana; https://www.purdue.edu/hhs/hsci/mrslab/basis_sets.html). In the current study, the “3T Siemens Difference Basis Set with Kaiser Coupling Constants,” were based on updated values for chemical shifts and J-GABA coupling constants (Kaiser et al., 2008; Kreis and Bolliger, 2012; Near et al., 2013). These slightly differ from to the originally generated basis sets, which used the values by Govindaraju et al. (2000). Based on the “edit off” spectra from the same MEGA-PRESS measurement and using the corresponding “3T Siemens Edit-off Basis set,” tNAA reference values for GABA+ and Glx were estimated. For most reliable quantitation results, the spline baseline constraint of “DKNTMN” within the LC-model routine was adapted. The DKNTMN parameter, which controls the minimum spacing between spline knots, plays a critical role in fitting the baseline curve and can significantly impact GABA+ and Glx quantification by influencing variance and potentially causing underestimation. To ensure precise measurements, we optimized this parameter, following a validated procedure (Koyun et al., 2024; Stock et al., 2023), within a range of 0.1 to 1.0 and ultimately set it to 0.45. This adjustment minimized the GABA+ measurement error while preserving the signal-to-noise ratio. Consistent application of this optimized value across all regions aligns with our previous methodologies, enhancing reliability and reducing potential biases in GABA+ and Glx assessment. To warrant adequate data quality, only spectra of final acceptable shim quality (FWHM of 3–7 Hz of the NAA peak) were used for the subsequent quantification. In the entire sample, we further assessed the absolute GABA+ error estimate, as this measure typically has a higher error than Glx or the reference metabolite. Doing so, we obtained values below the 15% Cramér–Rao lower bound (CRLB or %*SD*) criterion for all three VOIs.

The MRS data were collected prior to the MPH/placebo appointments on which participants performed the experimental task (also because MRS only allows to measure overall “baseline levels,” but not any changes in neurotransmission during task performance). MRS-assessed transmitter levels for the statistical analyses were not determined in an event-related fashion, hence reflecting baseline GABA+ and Glx levels in the VOIs. For the statistical analyses of the obtained data, an internal metabolite reference signal (Mikkelsen et al., 2017, 2019) was used. In this context, it is important that no systematic relationship between the reference metabolite and the parameter of investigation (here: response inhibition performance; Mikkelsen et al., 2019) exists. Given the absence of correlations between the relevant behavioral measure and tNAA (all *r* < 0.3; *p* > 0.2) (which minimizes, but never completely excludes the possibility that reported results are based on differences in the reference metabolite, rather than the investigated neurotransmitters), GABA+ and Glx were referenced in relation to tNAA (quantified from the edit-off spectra). A relative measure was formed by dividing GABA+/tNAA by Glx/tNAA to obtain a GABA+/Glx ratio. The resulting measures used for statistical analyses are ratios and therefore have no units.

### Experimental task

**Figure 2.**
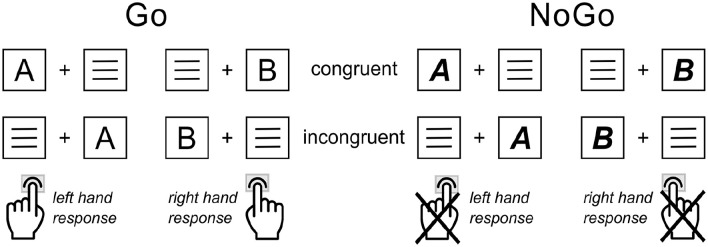
Simon NoGo paradigm: Illustrated are the possible stimulus-response configurations for the congruent/incongruent Go condition (left) and the congruent/incongruent NoGo condition (right). Congruency indicates the stimulus-response hand mapping of Go trials. Congruent trials required a Go response execution on the side the target letter stimulus was presented. In incongruent trials, the stimulus presentation side was opposite to the side of the correct Go response hand. The NoGo condition, as indicated by ***bold and italic*** target letter stimuli, required the participants to hold back any responses. Congruency was determined as for the Go trials.

A combined Simon and Go/NoGo paradigm (Chmielewski and Beste, 2017; Wendiggensen et al., 2022) was used to examine context-modulated response execution and inhibition. The task design and structure are depicted in Figure 2 and further detailed in the Supplemental Material.

### Statistical analysis: behavioral and MRS data

The behavioral data were analyzed using repeated measures ANOVAs with “experimental intervention” (MPH vs. placebo), “condition” (Go vs. NoGo), and “congruency” (congruent vs. incongruent) as within-subject factors. The between-subject factors were “MPH dose group” (low vs. high) and order of drug administration (MPH on first vs. second appointment). Significant main and interaction effects were examined with post hoc ANOVAs and/or post hoc *t-tests*. When a participant’s behavioral accuracy/performance was below chance level (<50%) in two or more task conditions, that case was marked as an outlier, and no longer considered for all subsequent analyses. Descriptive data are given as mean and standard error of the mean (SEM). Before running the ANOVAs, outliers regarding the MRS data were identified using the SPSS built-in exploratory outlier analysis. Whenever a case was identified as an extreme outlier (i.e., either 3rd quartile + 3*interquartile range, or 1st quartile—3*interquartile range) in a given MRS measure, that case was no longer considered representative of the sample and was excluded from all subsequent analyses.

To examine whether MRS-assessed transmitter levels correlated with task performance, linear correlation analyses (results in Table 1, step 1), as well as multiple linear regression analyses (results in Table 1, step 3), were performed. For that, the MRS-assessed transmitter levels were used as independent measures, and the relevant behavioral measure as the dependent variable. For a meaningful statistical comparison of the obtained correlation coefficients, we used Fisher’s *r*-to-*z* transformation method, testing the H_0_ that the two independent sample correlations are the same (Fisher, 1921; Weaver and Wuensch, 2013). This is achieved by transforming the correlation coefficients to *z*-scores (a common scale) and calculating the difference between them in terms of standard errors. Therefore, significant *p* values indicate that the two correlation coefficients are significantly different from each other (see Table 1, step 2). Whenever our a-priori hypotheses were directed, post hoc tests were conducted one-tailed.

**Table 1.** Results of statistical analysis of behavioral and ^1^H-MRS data.

Step 1. Pearson’s correlation of ^1^H-MRS measures and MPH-modulated response inhibition performance						Step 2. Fisher’s *r*-to-*z* transformation	
		Response inhibition performance on the MPH appointment					
		Low dose group		High dose group			
VOI	^1^H-MRS	*r*	*p*	*r*	*p*	*z*-score	*p*
Striatum	GABA+/Glx	0.549*	0.021	−0.107	0.358	1.699	*0.045*
	GABA+/tNAA	0.490*	0.038	−0.113	0.350	1.523	*0.064*
	Glx/NAA	−0.205	0.241	0.009	0.488	−0.509	*0.305*
SMA	GABA+/Glx	0.273	0.172	−0.710**	0.003	2.672	*0.004*
	GABA+/tNAA	0.090	0.379	−0.566*	0.022	1.675	*0.047*
	Glx/tNAA	−0.370	0.097	0.797**	<0.001	−3.384	*<0.001*
ACC	GABA+/Glx	−0.345	0.113	0.201	0.245	−1.322	*0.093*
	GABA+/tNAA	−0.394	0.082	0.315	0.136	−1.742	*0.041*
	Glx/tNAA	−0.026	0.465	−0.008	0.489	−0.042	*0.483*

*Note*. Step 1: Correlation significant (1-tailed): * at *p* < 0.05 level, ** at *p* < 0.01 level. Step2: Comparing the strength and confirming differences between correlation coefficients (*r*). Probability values (1-tailed) <0.05 indicate that correlation coefficients are significantly different from each other (bold font). ACC: anterior cingulate cortex; GABA+: gamma-aminobutyric acid plus macromolecules; Glx: glutamate-glutamine; ^1^H-MRS: proton-magnetic resonance spectroscopy; MPH: methylphenidate; tNAA: total N-acetylaspartate; SMA: supplementary motor cortex.

**Table:** 

Step 3. Regression coefficients for predicting the response inhibition performance stimulated with MPH										
Predictor	Low dose group					High dose group				
	*B*	*SE B*	*β*	*t*	*R* ^2^	*B*	*SE B*	*β*	*t*	*R* ^2^
Striatal GABA+/Glx	102.070	44.886	**0.549**	2.274	0.301*					
Constant	65.255	10.626		6.141						
Striatal GABA+/tNAA	129.853	66.628	**0.490**	1.949	**0.240**					
Constant	67.133	11.408		5.885						
SMA GABA+/Glx						−193.088	57.697	−**0.710**	−3.347	0.504*
Constant						130.419	14.502		8.993	
SMA GABA+/tNAA						−296.088	129.876	−**0.566**	−2.280	0.321*
Constant						131.869	21.831		6.041	
SMA Glx/tNAA						254.911	58.283	**0.797**	4.374	0.635*
Constant						−89.895	39.448		−2.279	

*Note*. Dependent variable: Response inhibition accuracy on the MPH appointment. *B* = unstandardized regression coefficient; *SE B* = standard error of the coefficient; *β* = standardized coefficient; *R*^2^ = goodness of fit (higher values indicate that a larger proportion of the variance in the dependent variable is explained by the independent variables). GABA+: gamma-aminobutyric acid plus macromolecules; Glx: glutamate-glutamine; MPH: methylphenidate; tNAA: total N-acetylaspartate; SMA: supplementary motor cortex.

* Marks significance at *p* < 0.05 level.

Given that non-signiﬁcant results obtained in regular parametric testing (including linear regression models) do not allow for reliable statements on whether or not the null hypothesis is more likely to be true than the alternative hypothesis, we further conducted Bayesian linear regression analyses for non-signiﬁcant results if there was a trend (*p* < 0.10) toward significance. Bayesian analyses were conducted to examine the relative evidence for the H_1_ compared to the *H*_0_. The Bayesian factor (BF_10_) with values higher than 3 provides moderate evidence for the *H*_1_ compared to the *H*_0_; BF_10_ values between 1 and 3 provide anecdotal evidence for the *H*_1_ to be true. On the other hand, BF_10_ values between 1/3—1 provide anecdotal evidence for the *H*_0_; while BF_10_ values below 1/3 indicate moderate evidence for the *H*_0_.

## Results

### Behavioral data

#### Task effects

Overall, the task-typical Simon effects (i.e., higher response accuracies in congruent trials in the Go condition and incongruent trials in the NoGo condition) were replicated (Chmielewski & Beste, 2017; Koyun et al., 2023; additional details are provided in the Supplemental Material).

#### MPH dose group effects

**Figure 3.**
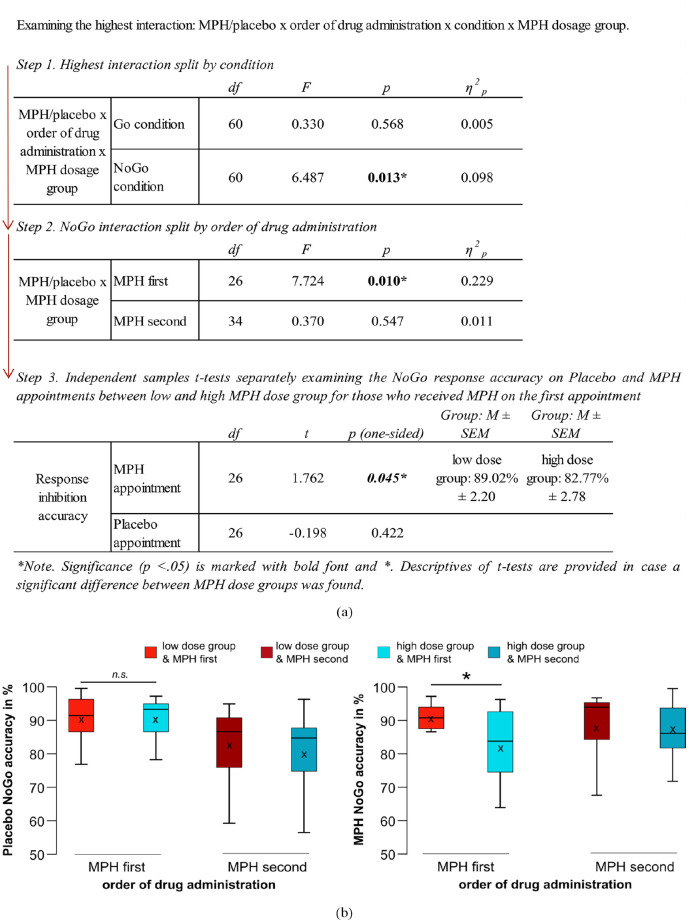
Behavioral results: (a) Summary of all post-hoc tests investigating the highest interaction. (b) Response inhibition accuracy. Depicted are boxplots illustrating the mean response inhibition accuracy for both MPH dose groups on the placebo (left plot) and the MPH appointment (right plot). Boxplots of the low dose MPH group are illustrated in nuances of red, and boxplots of those who received high doses of MPH are in nuances of blue. The *x*-axis denotes the order of drug administration, that is: MPH first = Participants received their MPH dose on the first appointment (and placebo on the second) and MPH second = Participants received their MPH dose on the second appointment (and placebo on the first). The “*x*” and the horizontal line inside the boxplots indicate the mean and median, respectively. Asterisks indicate significant differences at *p < 0.05* (one-tailed), and error bars represent the 95% confidence intervals. MPH: methylphenidate.

The repeated measures ANOVA for accuracy revealed an interaction of MPH/placebo × order of drug administration × condition × MPH dosage group (*F*_(1,60)_ = 6.692; *p* = 0.012; *η*^2^_*p*_ = 0.100). Post-hoc analyses separately examining the interaction effect in Go and NoGo conditions are summarized in Figure 3a. The performance (i.e., response accuracy) enhancement resulting from the administration of a low MPH dose on the first appointment was so substantial that it rendered the task experience/learning effect indiscernible in NoGo trials. The results are likely attributed to the near-optimal response inhibition performance of the low MPH dose group during the first appointment. Figure 3(b) visualizes the most relevant behavioral accuracy results.

The repeated measures ANOVA for the Go reaction times showed a main effect of MPH/placebo (*F*_(1,60)_ = 4.220; *p* = 0.044; *η*^2^_*p*_ = 0.066), indicating overall slightly faster responses when participants received MPH (454.24 ± 4.73 ms) as compared to the placebo (459.76 ± 5.06 ms). There was also an interaction of MPH/placebo x congruency x MPH dose group (*F*_(1,60)_ = 4.027; *p* = 0.049; *η*^2^_*p*_ = 0.063). Post hoc tests comparing the Go reaction times between the two dosage groups on the placebo and MPH appointments revealed no significant differences (all *p* > 0.546).

#### MRS-assessed transmitter levels as a predictor of response inhibition

Key differences in the behavioral response to low and high MPH dosage were mainly observed in response accuracy during the NoGo condition. Subsequent analyses focused on determining whether baseline GABA+ and Glx levels were associated with how the response inhibition performance was modulated by different MPH doses on the first appointment. Separate linear correlation analyses for each MPH dosage group were performed to investigate whether the MRS-assessed transmitter levels in the VOIs correlated with the response inhibition performance on the MPH appointment. These analyses revealed clear differences between the low and high MPH dosage groups (see Table 1). Specifically, only striatal MRS measures correlated with the response inhibition performance in the low dose group, while significant correlations were found only for MRS measures from the SMA in the high dose group.

Using Fisher’s *r*-to-*z* transformation, we determined whether the strength of the linear relationships (Step 1, *r*) significantly differed between the two dosage groups (see Table 1, Step 2). The results indicate that the linear correlation between response inhibition performance and striatal GABA+/Glx was higher in the low dose group. In contrast, the high dose group showed significantly stronger correlations between response inhibition and all MRS measures from the SMA. Additionally, a stronger relationship between response inhibition and GABA+/tNAA in the ACC was evident in the low dose group.

For a comprehensive understanding of the established linear correlations, linear regression analyses were performed to assess the significance of the relationship and magnitude of the effect. To examine whether the ^1^H-MRS-assessed transmitter levels were significant predictors, separate linear regression analyses were run for all ^1^H-MRS values that significantly correlated (confirmed with Fisher’s *r*-to-*z* transformation) with the response inhibition performance on the MPH appointment. A summary of all regression coefficients is provided in Table 1, Step 3.

**Figure 4.**
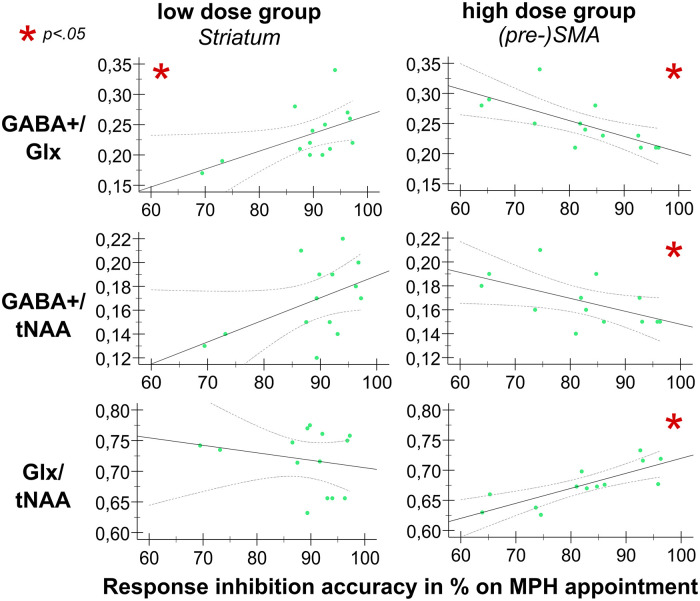
Relationship between ^1^H-MRS measures and response inhibition performance on the MPH appointment. Illustrated are scatter plots denoting the relationship between ^1^H-MRS measures and response inhibition performance for the low MPH dose group (left column) and high MPH dose group (right column), for the striatum and the (pre-)SMA, respectively. The *x*-axis denotes the mean response inhibition accuracy in % on the MPH appointment. The *y*-axis denotes the respective amino acid neurotransmitter levels. Significant correlations are marked with a red asterisk (*). ^1^H-MRS: proton-magnetic resonance spectroscopy; MPH: methylphenidate; SMA: supplementary motor cortex.

Figure 4 visualizes the main results of these analyses. For the low dose group, variations in mean response inhibition accuracy on the MPH appointment were significantly predicted by striatal GABA+/Glx concentrations (*F*_(1,12)_ = 5.171; *p* = 0.042), indicating that higher striatal GABA+/Glx levels are predictive of better inhibitory control in the low dose group. Additionally, there was a trend toward significance for striatal GABA+/tNAA as a predictor of response inhibition performance (*F*_(1,12)_ = 3.798; *p* = 0.075), but add-on Bayesian regression analysis indicated anecdotal evidence for the H_0_ (BF10 = 0.039).

For the high dose group, GABA+/Glx concentrations in the SMA significantly predicted variations in response inhibition performance stimulated with MPH (*F*_(1,11)_ = 11.199; *p* = 0.007), implying that response inhibition worsened if GABA+/Glx concentrations in the SMA were low. Moreover, both GABA+/tNAA and Glx/tNAA levels in the SMA significantly predicted response inhibition performance on the MPH appointment [(GABA+: (*F*_(1,11)_ = 5.197; *p* = 0.044); Glx: (*F*_(1,11)_ = 19.129; *p* = 0.001], indicating that in the high dose group, lower GABA+/tNAA levels in the (pre-)SMA were predictive of better inhibitory control when stimulated with MPH. Conversely, higher Glx/tNAA levels were associated with better response inhibition performance. Most of the variance observed in response inhibition was explained by Glx levels in the (pre-)SMA.

While all three predictors were statistically significant, the strongest association with response inhibition performance was observed for Glx/tNAA in the SMA (*β* = 0.797 as the largest standardized coefficient), implying that changes in Glx/tNAA levels in the (pre-)SMA have substantial effects (and overall largest influence) on response inhibition performance under high levels of catecholaminergic stimulation. Furthermore, GABA+/Glx (GABA+/Glx: *β* = −0.710) appears to have a stronger effect on response inhibition performance than GABA+/tNAA (*β* = −0.566) in the (pre-)SMA, suggesting that the inhibitory-excitatory ratio has a greater influence on/is more predictive of motor inhibitory accuracy than GABA+ concentrations alone.

## Discussion

We investigated the dynamic interplay between amino acid transmitters and catecholamines within the striatum, ACC, and (pre-)SMA and their predictive value on cognitive control processes. Specifically, we examined whether baseline GABA+ and Glx levels in key cortical networks of healthy adults are indicative of the magnitude of different MPH-dose-induced effects on response inhibition performance. Our results show that baseline GABA+/Glx concentrations in control-relevant regions are predictive of MPH modulations of response inhibition performance. Notably, the cortical region (and respective GABA+/Glx levels) associated with the MPH-induced effect on inhibitory control was MPH dose-specific. As hypothesized, administering low doses of MPH resulted in optimized performance, as evident at the first appointment. In this particular context, higher striatal GABA+/Glx levels were associated with better response inhibition performance. High doses of MPH resulted in comparably worse response inhibition performance, which was predicted by GABA+/Glx levels in the (pre-)SMA. We hence provide a first report of the predictive relevance of amino acid transmitter levels in control-relevant cortical regions for response inhibition performance under different degrees of catecholaminergic stimulation by MPH.

The behavioral data replicated the well-known task effects (Chmielewski and Beste, 2017; Koyun et al., 2023). Essentially, the most relevant behavioral differences between the two MPH dosage groups were in the response inhibition condition, which by task design is more demanding than the response execution condition. Prior studies with healthy adult samples also observed that the effects of MPH are most pronounced under increased task difficulty (where increased cognitive effort is needed to perform well) (Bensmann et al., 2019; Mückschel et al., 2020b). Here, significant differences between the low and high dose groups were evident, especially when participants received their respective MPH doses on the first appointment. Precisely, administering high MPH doses on the initial appointment resulted in significantly worse response inhibition performance as compared to the administration of low MPH doses. Of note, it is likely that the nearly optimal response inhibition performance exhibited by the low dose MPH group on the first appointment was significant enough to obscure any task experience/learning effects. The absence of interaction effects for those who received their respective MPH dose on the second appointment closely aligns with accounts suggesting that prior task experience significantly reduces or possibly eliminates MPH effects (Bensmann et al., 2019; Mückschel et al., 2020a, 2020b). Our findings suggest that task repetition may reduce the cognitive enhancements typically seen with MPH, potentially masking its effects once participants gain experience with the task. This may explain why dose-dependent effects of MPH on response inhibition were no longer detectable when task experience/familiarization was present. The proposed familiarity-induced diminishment of MPH effects highlights the importance of contextual factors in real-world academic or professional settings, where repeated exposure to similar tasks is common. Future research should explore how task learning (i.e., the reduction of task novelty and thus potentially effort, as well as the increase in response automatization) modulates MPH-induced effects across different cognitive domains, to delineate the boundaries of MPH’s cognitive-enhancing potential.

On the neurobiochemical level, the DA system in our healthy adult sample may have been relatively close to the optimal level (Linssen et al., 2014). Low doses of MPH thus likely induced catecholaminergic increases resulting in optimized motor inhibitory control performance (Arnsten and Dudley, 2005; Mehta et al., 2001). In line with the inverted *U*-shaped dose-response effect of MPH on inhibitory control processes (Tannock et al., 1995), high doses of MPH likely pushed participants’ catecholamine levels beyond optimum for the task at hand, leading to comparably worse response inhibition performance (Arnsten and Dudley, 2005; Berridge et al., 2006).

It was central to our study to investigate the extent to which the observed MPH dose-dependent effects were due to GABAergic and glutamatergic signaling in control-relevant cortical regions. We showed for the first time in a healthy adult sample that striatal GABA+/Glx levels are indicative of MPH-induced catecholaminergic effects on response inhibition. That is, higher striatal GABA+/Glx levels were predictive of low MPH dose-induced optimized response inhibition. Striatal GABA+/Glx ratios reflect the balance between inhibitory (GABA) and excitatory (glutamate) neurotransmission, which is essential for cognitive control and response control processes (Beste et al., 2008; de la Vega et al., 2014; Purkayastha et al., 2015). MPH treatment is known to increase DA and NE availability (Faraone, 2018; Volkow et al., 2001) and has been shown to modulate GABAergic and glutamatergic signaling in the striatum (Valjent et al., 2019). The present results suggest the GABA+/Glx ratio as critical marker for optimal striatal functioning under low-dose catecholaminergic stimulation, offering greater sensitivity than GABA+ and Glx levels alone. Therefore, examining neurotransmitter ratios in certain cortical areas may provide a more comprehensive understanding of the neurochemical dynamics underlying cognitive control processes than evaluating individual neurotransmitter levels. Our results partially indicate the involvement of the striatal GABAergic system in response selection and inhibition through GABAergic neural transmission (Bar-Gad et al., 2003; Quetscher et al., 2015). GABA is abundant in striatal structures and plays a crucial role in the functioning of MSNs. Striatal MSNs are assumed to form a winner-takes-all network (WTA; Plenz, 2003), in which inhibitory interconnections among MSNs inhibit nearby neurons, thereby suppressing competing actions and causing the network to converge on a sole “winner.” MPH-induced DA increases likely induce increased GABA release from striatal interneurons (e.g., Freese et al., 2012; Tritsch and Sabatini, 2012). This distinctive WTA may be strengthened by elevated levels of GABA in the striatum, leading to more robust suppression of prepotent responses/alternative action plans (Yildiz et al., 2014). Catecholamines, especially DA, play crucial roles in modulating the strength of connections within the WTA in the striatum. Notably, DA-glutamate interactions are present on/innervate approximately 95% of striatal neurons (particularly MSNs), and heavily modulate each other’s function and release (Lorenz et al., 2015; Nair et al., 2014). DA modulates glutamatergic neurons in striatal structures and finely tunes their sensitivity to glutamatergic input from the PFC (Nair et al., 2014). PFC glutamatergic afferents were shown to play a key role in acute effects of MPH (Wanchoo et al., 2009), whereas glutamatergic signaling in the caudate nucleus appeared to be crucial for chronic, but not necessarily for acute effects of MPH (Nair et al., 2014). Taken together, our low MPH dose-induced increases in catecholamines may have further facilitated/improved the striatum’s role in adjusting response caution (Forstmann et al., 2008), allowing for optimal motor response inhibition performance in high response tendency scenarios.

Interestingly, lower GABA+/Glx baseline concentrations in the (pre-)SMA (and not the striatum) were predictive of better motor response inhibition performance when participants received high doses of MPH on the first appointment. The SMA is directly connected to the motor system and plays a crucial role in adaptively adjusting motor behavior (Nachev et al., 2007; Shirota et al., 2019). Elevating the catecholaminergic levels beyond the optimum likely increased locomotor activity and most importantly automatic/prepotent response tendencies, necessitating additional motor/cognitive control and inhibition by the SMA (Berridge et al., 2006; Hermans et al., 2018). Regarding the neurochemical properties within the (pre-)SMA, GABA and glutamate have been suggested to play roles in reactive and proactive inhibition respectively (Hermans et al., 2018; Weerasekera et al., 2020). In our sample, higher Glx levels in the (pre-)SMA were associated with effective response inhibition performance under high doses of MPH. Despite the present modulation of the catecholaminergic systems, the results are in line with previous reports from MRS in healthy adults (Weerasekera et al., 2020). The complex interplay between Glx and catecholaminergic neurotransmission suggests that the MPH effect in response inhibition may be influenced by the duration of catecholaminergic modulation and the specific receptor subtypes involved (Caravaggio et al., 2016). The acute stimulation of catecholaminergic signaling as induced by high doses of MPH may have triggered compensatory mechanisms or receptor-specific effects, particularly involving NE adrenoceptors and DA D1/D2 receptors in the PFC, to maintain cognitive control under excessive DA/NE levels (Arnsten and Dudley, 2005; Caravaggio et al., 2016). Additionally, high dose MPH-induced hyperlocomotion, possibly linked to suppression of PFC glutamatergic transmissions (Cheng et al., 2014), may reflect the delicate balance of catecholaminergic activity and Glx-driven excitatory control. Thus, if high doses of MPH indeed suppressed PFC glutamatergic transmission, then higher Glx levels in the (pre-)SMA may facilitate signaling in the pathway mediating cognitive/inhibitory control (through compensatory mechanisms), thereby improving motor response inhibition. Additionally, participants with lower levels of GABA+ in the SMA, possibly indicative of less inhibitory control (Naaijen et al., 2017; Puts, 2020), showed a greater behavioral benefit from high MPH doses compared to those with higher GABA+ levels. Clearly, the (pre-)SMA dynamically regulates motor responses by balancing GABAergic inhibition and glutamatergic excitation, allowing adjustments of movements in accordance with behavioral goals. Our results indicate that the baseline neurochemical balance of GABA+ and Glx (partially) underlies the (pre-)SMA’s role in successful response inhibition when stimulating the catecholaminergic system with high doses of MPH.

In the ACC, higher GABA levels have been associated with better response inhibition performance (Silveri et al., 2013) as well as conflict monitoring and control (Takacs et al., 2021). The previously established relationship appears not to persist when the catecholaminergic system has been modulated with MPH, further underlining the complex and modulation-sensitive interplay of amino acid transmitters and catecholamines within cortical networks. Alternatively, it has been suggested that the ACC is especially related to response selection and successful execution rather than motor inhibition per se, which may underlie the lack of correlations between response inhibition performance and MRS measures from the ACC we observed (Adams et al., 2017; Takacs et al., 2021).

Taken together, GABA+ and Glx interact with the catecholaminergic system through complex and specific molecular and synaptic mechanisms that regulate cognitive control. DA, acting via D1 and D2 receptors, differentially modulates GABAergic and glutamatergic signaling, with D1 receptor activation likely enhancing N-methyl-D-aspartate (NMDA) receptor-mediated activity, thereby increasing excitatory signaling, and D2 receptors adjusting GABA+ release, thereby fine-tuning inhibitory control (Ampe et al., 2007). Especially in the striatum, dopamine has further been shown to modulate the sensitivity of GABAergic MSNs to the glutamatergic input from the PFC and thalamus, which should directly influence response selection and inhibition processes (Brodal, 2010; Burke et al., 2017; Stock et al., 2023). Complementing this, NE, acting via adrenergic α2A receptors, enhances cognitive control in the PFC and connected cortical hubs by modulating GABA+ and Glx (Salgado et al., 2012; Zhang et al., 2013). MPH-induced increases in catecholamine levels likely amplify these receptor-mediated interactions to optimize cognitive processes at lower doses. However, at higher doses, MPH may overactivate these pathways, thereby disrupting the balance of excitatory/inhibitory control and impairing cognitive function, possibly through altered neurotransmitter reuptake mechanisms.

The implication of the results provided in this study is that individual differences in amino acid transmitter levels, such as GABA+, Glx, and their ratio, may influence MPH efficacy. Specifically, baseline amino acid transmitter levels appear to significantly affect responses to pharmacological interventions like MPH administration, indicating that different brain regions may support similar cognitive processes based on interindividual neurochemical states. These individual neurochemical profiles could serve as biomarkers for more personalized, targeted treatments, and thereby potentially also inform future research. Additionally, the current findings highlight the importance of exploring how task experience and neurochemical variability jointly modulate MPH-induced effects across different cognitive domains.

With respect to methodological limitations, future MRS studies on this topic should also include a water-unsuppressed acquisition for normalization of the spectra to provide another option for the normalization of the spectra.

## Conclusion

In line with prior studies of our group, MPH dose-dependent effects were present especially on the initial appointment (when no task experience existed). In this particular context, the effects of MPH exhibited an inverted *U*-shaped function, wherein low MPH doses optimized inhibitory control, while high doses of MPH resulted in comparably worse response inhibition performance. Furthermore, MPH dose-dependent effects were no longer discernible when participants were already familiar with the task at hand, suggesting that prior task experience likely masks MPH effects. Most importantly, we showed a predictive relationship of baseline GABA+ and Glx in the striatum and (pre-)SMA of healthy adults onto the modulatory effects of MPH on (motor) inhibitory control. Specifically, the beneficial effects of low MPH doses on response inhibition performance were associated with higher baseline striatal GABA+/Glx concentrations, whereas the impeding effects of high MPH doses on motor inhibitory control were predicted by baseline GABA+/Glx, especially Glx, levels in the (pre-)SMA.

## Supplemental Material

sj-pdf-1-jop-10.1177_02698811251340893 – Supplemental material for GABA and Glx levels in cortico-subcortical networks predict catecholaminergic effects on response inhibition Supplemental material, sj-pdf-1-jop-10.1177_02698811251340893 for GABA and Glx levels in cortico-subcortical networks predict catecholaminergic effects on response inhibition by Anna Helin Koyun, Annett Werner, Paul Kuntke, Veit Roessner, Christian Beste and Ann-Kathrin Stock in Journal of Psychopharmacology
