# Supplementary material for: GABA and Glx levels in cortico-subcortical networks predict catecholaminergic effects on response inhibition
Source: J Psychopharmacol. 2025 Jun 20;39(8):769–81. doi: 10.1177/02698811251340893 (PMC12287562; doi:10.1177/02698811251340893)
Supplement: sj-pdf-1-jop-10.1177_02698811251340893 – Supplemental material for GABA and Glx levels in cortico-subcortical networks predict catecholaminergic effects on response inhibition [file sj-pdf-1-jop-10.1177_02698811251340893.pdf]

Supplementary materials

Publication title: ***GABA and Glx levels in cortico-subcortical networks predict catecholaminergic effects on response inhibition***

Authors: *Anna Helin Koyun, Annett Werner, Paul Kuntke, Veit Rößner, Christian Beste, Ann-Kathrin Stock*

Additional details on participant exclusion decision

In total, n = 14 participants were excluded from subsequent analyses due to the following reasons: n = 1 dropped out after the first appointment, for n = 5 response accuracy was below chance level (< 50%) in at least two task conditions, there were technical problems during experimental task performance for n = 1, n = 4 participants were identified as extreme outliers regarding the MRS data, and n = 3 were excluded due to unquantifiable MRS data due to poor signal quality (e.g., due to movement artifacts, poor shimming).

Task description

Each trial began with the simultaneous presentation (for 200 ms) of a single target letter stimulus (i.e., a yellow letter "A" or "B") and a contralateral distractor stimulus (three horizontal white lines matching the letter stimulus in size) within the two white frame boxes. The font of the letter stimulus indicated whether the current trial was a Go trial (normal font) or a NoGo trial (bold-italic font). In response to the letter stimulus "A" in normal font, participants were instructed to respond by pressing the left "Ctrl" button with their left index finger (and the right "Ctrl" button with their right index finger when it was a "B") (Go; 70% of all trials). This rule applied regardless of the spatial location (left or right-hand side) the target letter stimulus was presented on. When the letter stimulus was presented in a bold-italic font (i.e., a yellow "***A***" or "***B***"), participants had to withhold all motor responses (NoGo; 30% of all trials). In case the letter stimulus was presented at the spatially opposite location regarding the associated Go response hand (i.e., "A" or "***A***" presented on the right, "B" or "***B***" presented on the left), the trials were coded as incongruent trials. The combination of letter stimulus and spatial location results in the following four conditions: (1) congruent Go trials, (2) incongruent Go trials, (3) congruent NoGo trials, and (4) incongruent NoGo trials. Trial congruency in both Go and NoGo conditions was randomly varied, with congruent and incongruent trials being presented at the same frequency (50%) in each condition. Responses to Go trials within 1700 ms after stimulus presentation were coded as "correct" or "incorrect". A speed-up sign (i.e., "Schneller!" / "Faster!") was presented above the fixation cross in case no response was given within 500 ms after stimulus onset in Go trials. Depending on the condition, trials in which no response was obtained post-stimulus presentation were either coded as Go trial "misses" or as NoGo trial "correct omissions". In NoGo conditions, any response obtained within 1700 ms after stimulus presentation was coded as a "false alarm". The inter-trial interval (ITI) was jittered between 1300 and 1700 ms. The experimental paradigm consisted of 6 blocks with 120 trials each, resulting in a total number of 720 trials.

At the first appointment following the administration of MPH/placebo, participants completed a practice session consisting of 16 trials to familiarize themselves with the task. Participants were instructed to respond as fast and accurate as possible, aiming to minimize occurrences of the "Faster!" speed-up sign. After each block (120 trials), participants could take a self-timed break (i.e., to rest eyes) before resuming the task with a button press. The experiment took approximately 30 minutes to complete.

Typical task effects

Overall, the task-typical Simon effects (i.e., better performance in congruent trials in the Go condition, and better performance in incongruent trials in the NoGo condition) were replicated [1,2]. The ANOVA showed a main effect of condition ($F_{(1,60)}$ =109.691; p <.001; η^2_p = 0.646), with overall higher accuracy in Go (95.65 % ± 0.41) than in NoGo trials (86.27 % ± 1.08). There was also a main effect of congruency ($F_{(1,60)}$ = 20.872; p <.001; η^2_p = 0.258), showing higher accuracy in incongruent (91.71 % ± 0.70) than in congruent trials (90.21 % ± 0.71). Importantly, there was an interaction of trial congruency x condition ($F_{(1,60)}$ = 70.385; p <.001; η^2_p = 0.540). Post-hoc paired t-tests confirmed opposing congruency effects in Go trials ($t_{(63)}$ =4.268; p<.001; congruent = 96.55 % ± 0.38; incongruent = 94.54 % ± 0.55) and NoGo trials ($t_{(63)}$ =-8.760; p <.001; congruent = 83.43 % ±1.20; incongruent = 88.65 % ± 1.03).

Additional MRS Data

In the following, representative LCModel fits (red line) of MEGA-PRESS for the three VOIs (striatum, SMA, and ACC) are provided. Upper parts: Residual curve (depicting the difference between the fitted and the measured curves). Lower parts: black curves represent the measured spectrum and the baseline. On the right are metabolite estimates (in arbitrary units). GABA+: GABA and macro molecules, Glx: combination of glutamate and glutamine, NAA: N-Acetyl-aspartate.

Figure S1 a. *Representative LCModel fit of MEGA-PRESS for the striatum.*

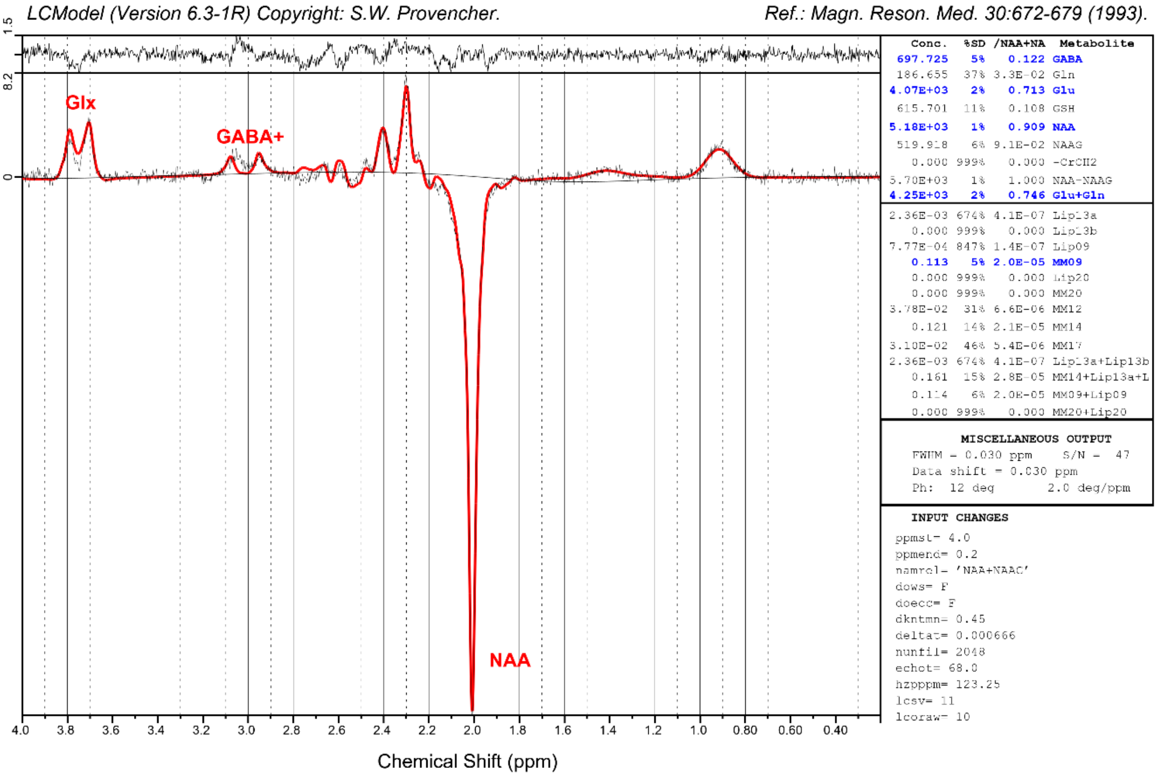

Figure S1 b. *Representative LCModel fit of MEGA-PRESS for the SMA.*

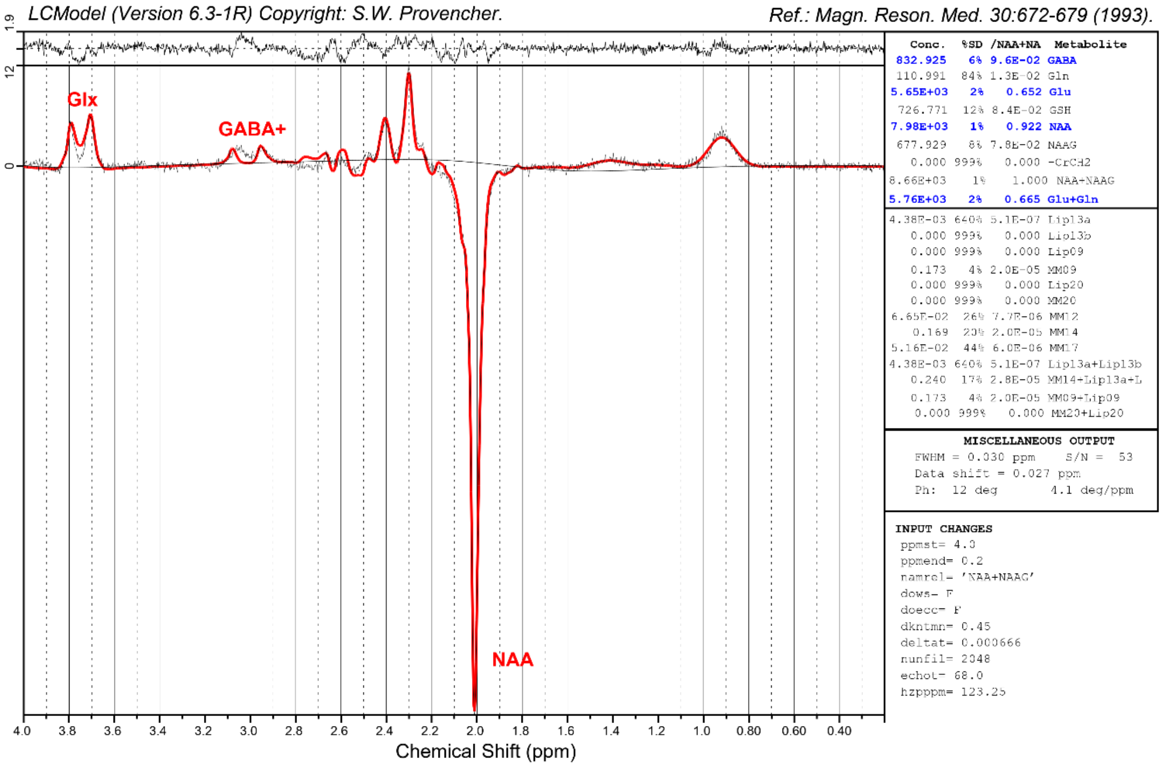

Figure S1 c. *Representative LCModel fit of MEGA-PRESS for the ACC.*

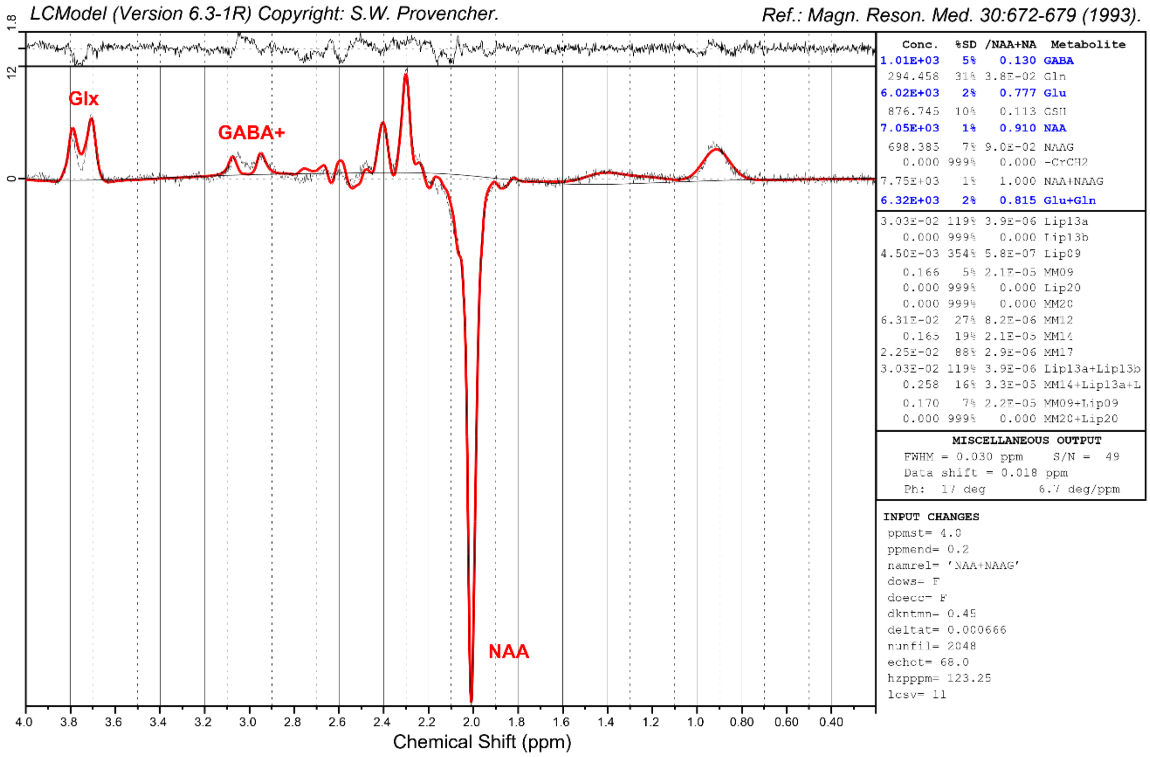

***Table S1** Regional Tissue Composition in low and high MPH dose groups: Overview of CSF, Grey Matter, and White Matter Fractions across ACC, SMA, and Striatum*

MPH dose group	VOI	Tissue	Fraction Mean	Fraction SD
**low dose group**	ACC	fCSF	0.13681	0.02918
		fGM	0.52619	0.04714
		fWM	0.3371	0.03842
	SMA	fCSF	0.18753	0.0424
		fGM	0.48283	0.0496
		fWM	0.3297	0.04702
	striatum	fCSF	0.07168	0.02229
		fGM	0.56781	0.03264
		fWM	0.36039	0.04637
**high dose group**	ACC	fCSF	0.13219	0.03412
		fGM	0.52441	0.0502
		fWM	0.34334	0.04812
	SMA	fCSF	0.20723	0.09706
		fGM	0.49137	0.04571
		fWM	0.30123	0.06719
	striatum	fCSF	0.06913	0.029
		fGM	0.5615	0.06156
		fWM	0.36938	0.07482

The table presents the mean and standard deviation (SD) of tissue fractions (fractions of cerebrospinal fluid (fCSF), grey matter (fGM), and white matter (fWM)) for the three volumes of interest (VOIs: ACC, SMA, and striatum) by MPH dose group (low dose group and high dose group).

***Table S2** Full Width at Half Maximum (FWHM) and Signal-to-Noise Ratio (SNR) for each included participant (final sample n =64) and Volume of Interest (VOI). The table presents the FWHM and SNR values calculated for all included participants across the defined VOIs. Each row corresponds to an individual participant, with separate columns detailing the FWHM and SNR for each VOI. This data provides insight into the image quality and reliability of the measured signals in the specified regions.*

	**FWHM (ppm)**			**SNR**		
**side**	**ACC**	**SMA**	**striatum**	**ACC**	**SMA**	**striatum**
**Participant numbering (#)**						
**002**	0,029	0,033	0,033	39	45	44
**003**	0,038	0,038	0,043	41	46	41
**005**	0,029	0,033	0,057	39	44	43
**006**	0,033	0,043	0,038	46	49	40
**007**	0,029	0,048	0,038	48	44	39
**009**	0,029	0,043	0,043	44	44	41
**010**	0,029	0,038	0,033	32	43	42
**011**	0,033	0,043	0,033	42	49	40
**012**	0,033	0,048	0,033	40	43	44

**013**	0,029	0,033	0,038	37	44	42
**015**	0,029	0,029	0,033	41	41	37
**016**	0,033	0,033	0,038	38	43	43
**017**	0,038		0,057	38		48
**018**	0,033	0,033	0,043	34	42	41
**019**	0,033	0,033	0,033	40	45	43
**022**	0,033	0,043	0,038	38	46	42
**023**	0,029	0,038	0,033	41	42	41
**024**	0,024	0,048	0,038	41	45	46
**026**	0,038	0,038	0,033	40	46	43
**027**	0,029	0,038	0,033	39	48	41
**028**	0,033	0,033	0,038	36	44	45
**029**	0,128		0,043	53		44
**030**	0,024	0,033	0,033	37	43	40
**031**	0,029	0,043	0,033	41	45	41
**032**	0,033	0,038	0,043	37	44	41
**033**	0,033	0,057	0,043	41	38	41
**034**	0,033	0,029	0,043	39	47	46
**035**	0,029	0,033	0,048	40	45	52
**036**	0,029	0,038	0,038	41	35	38
**037**	0,033	0,038	0,033	41	51	44
**039**	0,033	0,033	0,043	40	47	46
**040**	0,029	0,033	0,038	38	49	42
**041**	0,029	0,029	0,038	41	47	47
**042**	0,033	0,029	0,043	32	48	48
**043**	0,029	0,043	0,038	38	45	44
**044**	0,033	0,029	0,043	42	47	45
**045**	0,033	0,043	0,038	40	54	43
**046**	0,038	0,043	0,038	48	51	44
**047**	0,038	0,048	0,076	43	42	46
**048**	0,214	0,043	0,048	128	44	47
**049**	0,029	0,043	0,043	38	48	42
**050**	0,029	0,048	0,038	38	44	51
**051**	0,038	0,033	0,038	41	47	42
**052**	0,033	0,033	0,043	36	46	45
**053**	0,048		0,052	53		50
**055**	0,038	0,038	0,043	41	45	43
**056**	0,029	0,038	0,043	39	45	43
**057**	0,033	0,038	0,048	39	45	45
**058**	0,033	0,057	0,038	12	44	29
**059**	0,057		0,057	41		52
**060**	0,029	0,043	0,038	38	48	45
**061**	0,033	0,048	0,043	41	52	48
**062**	0,033	0,048	0,048	41	51	49
**063**	0,038	0,029	0,038	37	46	43
**064**	0,043	0,033	0,038	36	50	44

**065**	0,038	0,038	0,043	43	43	42
**068**	0,029	0,033	0,038	38	48	45
**069**	0,029	0,038	0,033	40	39	39
**070**	0,033	0,043	0,038	41	48	47
**071**	0,029	0,038	0,038	41	50	43
**072**	0,033	0,043	0,038	43	46	47
**074**	0,033	0,033	0,043	35	43	46
**075**	0,033	0,043	0,038	38	55	43
**076**	0,029	0,038	0,048	39	47	44

Table S3 Supplementary MRS Data. *This table presents the MRS data for all participants (n = 78) of this study. Metabolite concentrations for GABA+, Glx, and tNAA are reported alongside their corresponding standard deviations (SD). Additionally, participant-specific information is provided, including participant number (#), pharmacological group assignment, and inclusion/exclusion status.*

			**ACC**						**SMA**						**Striatum**					
**#**	**pharm.group**	**inclusion decision**	**GABA+**	**SD**	**Glx**	**SD**	**tNAA**	**SD**	**GABA+**	**SD**	**Glx**	**SD**	**tNAA**	**SD**	**GABA+**	**SD**	**Glx**	**SD**	**tNAA**	**SD**
001	high MPH group	*excluded*																		
002	high MPH group	included	902.564	5	4710	4	5660	1	1120	5	4940	5	6720	2	1140	6	4220	5	7070	1
003	low MPH group	included	794.08	6	4570	4	5530	1	962.754	5	3750	5	5230	1	646.186	6	2450	5	3460	2
004	high MPH group	*excluded*																		
005	high MPH group	included	672.551	6	3450	5	4240	2	666.117	7	3270	5	4430	2	781.352	5	2020	8	3030	2
006	low MPH group	included	890.263	6	6250	5	7570	1	1200	6	4560	5	6620	1	668.578	6	2880	5	3930	1
007	low MPH group	included	844.328	6	5300	5	6740	1	1130	5	4330	5	6120	2	788.682	5	2140	7	3570	2
008	high MPH group	*excluded*	1210	5	5830	5	7230	1	1330	5	4800	5	6630	2	765.542	6	3640	5	5020	1
009	low MPH group	included	856.362	6	5430	5	6590	1	973.447	5	4240	5	5600	1	678.197	6	2330	6	3450	2
010	high MPH group	included	1040	6	5810	4	7360	1	1010	6	4950	5	7030	1	696.922	6	3120	5	4410	1
011	low MPH group	included	1050	6	5310	4	6100	1	1420	5	4690	5	6710	2	719.91	6	3670	5	5150	1
012	high MPH group	included	829.89	6	4890	4	5830	1	898.777	6	3470	5	4700	1	898.917	5	3330	5	4670	1
013	high MPH group	included	692.554	6	4430	5	5040	1	971.973	6	4200	5	5820	2	641.018	7	3000	5	3810	1
014	low MPH group	*excluded*	716.807	6	3880	5	4970	1	732.202	6	3860	5	5490	1	684.534	6	3220	5	4380	1
015	low MPH group	included	613.7	7	3690	5	4520	1	611.384	6	2860	5	3910	1	640.668	6	2510	5	3530	2

			**ACC**						**SMA**						**Striatum**					
**#**	**pharm.group**	**inclusion decision**	**GABA+**	**SD**	**Glx**	**SD**	**tNAA**	**SD**	**GABA+**	**SD**	**Glx**	**SD**	**tNAA**	**SD**	**GABA+**	**SD**	**Glx**	**SD**	**tNAA**	**SD**
016	low MPH group	included	940.298	5	5040	4	6040	1	878.335	6	4420	4	5970	1	720.754	6	3290	5	4310	1
017	low MPH group	included	865.511	6	4950	5	5910	1							809.734	6	3290	5	4610	1
018	low MPH group	included	790.891	5	4410	4	5030	2	742.999	6	4330	5	5930	1	738.569	6	2970	5	4100	1
019	low MPH group	included	962.92	5	5450	4	6610	1	1030	6	4790	5	7230	1	710.348	6	3490	5	5130	1
020	low MPH group	*excluded*	1750	6	9750	5	12100	1	2010	6	10100	5	14400	1	1250	7	6360	5	8350	1
021	low MPH group	*excluded*	848.306	6	4560	4	4930	1	894.968	6	4550	5	6750	1	676.618	6	3220	5	4230	1
022	high MPH group	included	1050	5	5320	4	6570	1	1410	5	4570	5	7110	2	757.31	6	3570	5	5070	1
023	low MPH group	included	840.582	6	4760	5	6270	1	951.655	5	3470	5	5680	1	708.157	6	3110	5	4750	1
024	high MPH group	included	760.678	6	4710	4	5470	1	935.501	6	3530	5	4970	2	675.127	6	3170	5	4450	1
025	low MPH group	*excluded*	968.28	5	3650	5	4060	2							854.459	5	2890	6	4030	2
026	high MPH group	included	766.065	6	4240	4	4830	1	861.101	6	4230	5	5930	1	643.032	6	3000	5	4260	1
027	high MPH group	included	920.838	6	5070	4	6180	1	1010	5	4450	5	6270	1	837.275	5	3430	5	4550	1
028	high MPH group	included	816.018	6	4280	4	5160	1	928.974	5	4030	5	5700	1	641.059	7	3160	5	4530	1
029	high MPH group	included	1080	5	4860	5	6110	1							791.711	5	2850	5	4180	1
030	high MPH group	included	943.104	5	4870	4	5440	2	978.687	6	5100	4	6950	1	615.168	6	2920	5	4030	1
031	high MPH group	included	855.624	6	4660	4	5870	1	1260	5	3960	6	5890	2	121.578	8	473.942	5	688.939	1
032	high MPH group	included	664.807	6	3720	5	4860	1	850.108	6	3510	5	5040	1	650.364	6	2600	5	3750	1

			ACC						SMA						Striatum					
**#**	**pharm.group**	**inclusion decision**	**GABA+**	**SD**	**Glx**	**SD**	**tNAA**	**SD**	**GABA+**	**SD**	**Glx**	**SD**	**tNAA**	**SD**	**GABA+**	**SD**	**Glx**	**SD**	**tNAA**	**SD**
033	high MPH group	included	909.608	5	4430	5	5690	1	793.864	6	3600	5	5230	1	858.175	5	2870	5	4230	1
034	high MPH group	included	842.042	5	4510	4	5340	1	748.79	7	4140	5	6400	1	1040	5	3320	5	4640	1
035	low MPH group	included	846.905	6	5150	5	6340	1	950.847	6	4190	5	6200	1						51
036	high MPH group	included	626.87	6	3740	5	4350	1	530.984	7	2690	6	3430	2	632.868	6	2510	5	3300	1
037	low MPH group	included	952.144	6	5070	4	6220	1	1100	5	5130	5	6960	1	753.453	6	3430	4	5000	1
038	low MPH group	*excluded*	945.698	6	5510	4	6270	1	1070	6	4270	5	6390	1	663.547	7	3710	5	5220	1
039	high MPH group	included	815.265	6	4490	4	5160	1	850.578	6	4090	5	5670	1	712.097	6	3210	5	4640	1
040	low MPH group	included	869.359	6	5110	5	6590	1	832.925	6	5000	5	7030	1	711.917	6	2840	5	4260	1
041	low MPH group	included	911.849	5	4340	5	5900	1	898.546	6	4050	4	6680	1	776.16	5	2880	5	4290	1
042	high MPH group	included	834.132	6	5000	4	5660	1	965.033	6	4800	5	6930	1	680.752	7	3720	5	5170	1
043	high MPH group	included	755.808	6	4250	5	5180	2	955.094	6	3420	5	5130	2	620.164	6	2700	5	4200	1
044	low MPH group	included	903.839	6	5000	4	6360	1	982.304	5	4380	5	6430	1	688.388	6	3010	5	4500	1
045	high MPH group	included	873.404	5	4870	4	5870	1	1140	5	4800	5	7140	1	603.981	6	3020	5	4450	1
046	low MPH group	included	958.903	6	5780	5	7310	1	1250	5	4750	5	7030	1	758.103	6	3540	5	5170	1
047	low MPH group	included	1460	7	7050	5	9370	1	1990	6	6950	6	10500	1	1430	6	4590	7	6900	2
048	low MPH group	included	725.785	6	3990	5	4850	1	629.004	6	2430	5	3360	1	616.178	6	3120	5	4940	1
049	high MPH group	included	966.566	6	5410	4	6810	1	1110	6	5000	5	7410	2	714.021	6	2960	5	4550	1

			ACC						SMA						Striatum					
**#**	**pharm.group**	**inclusion decision**	**GABA+**	**SD**	**Glx**	**SD**	**tNAA**	**SD**	**GABA+**	**SD**	**Glx**	**SD**	**tNAA**	**SD**	**GABA+**	**SD**	**Glx**	**SD**	**tNAA**	**SD**
050	low MPH group	included	841.386	6	4430	5	5770	1	831.445	6	4230	5	6030	1	693.093	7	3970	5	5500	1
051	high MPH group	included	775.528	5	3850	4	4700	1	658.738	6	3170	5	4800	1	546.134	7	2640	6	3590	2
052	high MPH group	included	755.774	6	4130	5	5430	1	763.349	6	4470	5	6020	1	783.175	5	3210	5	4290	1
053	low MPH group	included	887.32	5	4480	5	5130	1	634.047	6	3020	5	3940	1	598.089	7	3340	5	4620	1
054	low MPH group	*excluded*	1930	5	8580	5	10100	2	1590	7	7700	5	11400	1	1300	7	4470	7	6760	2
055	low MPH group	included	824.421	5	3780	5	4370	1	677.087	6	3610	5	5130	1	633.811	6	2960	5	4090	1
056	low MPH group	included	749.961	6	4230	5	5030	1	820.261	6	3590	5	5100	1	755.875	6	2740	5	3660	2
057	low MPH group	included	770.137	6	3990	4	4400	1	744.105	6	3460	5	4910	1	651.035	6	2680	5	3710	1
058	high MPH group	included	876.28	6	5480	11	7210	3	799.303	7	4420	14	7310	3	602.12	7	3080	15	4830	3
059	high MPH group	included	1100	5	4910	4	5660	1							799.785	6	2870	6	4540	1
060	low MPH group	included	809.175	6	4130	5	5040	1	901.579	5	4120	5	5530	1	641.248	6	3180	5	4570	1
061	high MPH group	included	779.055	6	4250	5	5190	1	942.01	5	3770	4	5350	1	642.123	7	2970	5	4160	1
062	high MPH group	included	912.634	5	4390	5	5340	1	1090	5	4360	5	6360	1	849.173	5	2880	5	4650	1
063	low MPH group	included	831.163	6	4690	4	5930	1	801.649	6	4270	4	6570	1	784.26	5	3180	5	4180	1
064	low MPH group	included	926.722	5	5330	4	6240	1	922.637	6	4520	5	6890	1	718.066	6	3330	5	4830	1
065	high MPH group	included	1040	6	5700	4	6680	1	1060	5	4500	4	7130	1	720.129	6	2910	5	4430	1
066	low MPH group	*excluded*																		

			ACC						SMA						Striatum					
**#**	**pharm.group**	**inclusion decision**	**GABA+**	**SD**	**Glx**	**SD**	**tNAA**	**SD**	**GABA+**	**SD**	**Glx**	**SD**	**tNAA**	**SD**	**GABA+**	**SD**	**Glx**	**SD**	**tNAA**	**SD**
067	low MPH group	*excluded*	1010	5	5560	4	6520	1	11.745	56	155.143	12	259.575	2	731.496	6	2870	5	4210	1
068	high MPH group	included	758.186	6	4800	5	6070	1	1050	6	4620	5	6850	1	818.722	6	2710	5	4170	1
069	low MPH group	included	852.672	6	4890	5	6020	1	808.988	6	3620	5	5020	1	735.953	6	3070	5	4270	2
070	high MPH group	included	936.094	6	5190	4	6190	1	1080	5	4160	5	6170	1	745.68	6	2920	5	4020	1
071	low MPH group	included	905.175	5	5070	4	5960	1	887.496	6	3960	5	5950	1	781.289	6	3110	5	4000	2
072	high MPH group	included	694.91	6	4030	5	5420	1	912.654	5	3550	5	5520	1	619.906	7	3030	5	4530	1
073	low MPH group	*excluded*																		
074	high MPH group	included	896.43	5	4310	5	5510	2	824.995	6	4100	5	5790	1	845.883	5	2920	5	4070	2
075	low MPH group	included	787.147	6	3790	5	4800	1	820.342	6	3580	5	5510	1	747.689	5	2840	5	4210	2
076	low MPH group	included	799.221	6	4530	4	5230	1	930.776	5	3960	5	5750	1	730.438	5	2950	5	4020	1
077	high MPH group	*excluded*	1040	6	5900	4	7030	1			0	999	0	999	750.246	6	3330	5	4590	1
078	low MPH group	*excluded*	872.384	6	4730	4	5590	1	954.22	6	4340	5	5690	1	803.91	6	2960	5	4160	1

Table S4 Comparison of baseline MRS metrics between pharmacological groups. This table summarizes the results of independent samples t-tests comparing the above-provided MRS data from Tables S1-3 between the two pharmacological groups (low vs. high MPH group) in the final sample. The analyses included metabolite concentrations (GABA+, Glx, tNAA), fractions (white matter ,grey matter, CSF), and data quality measures (FWHM, SNR). Degrees of freedom (df) and t-test statistics are provided for each comparison, along with p-values indicating statistical significance. These results show no relevant group differences in MRS-derived baseline measures, fractions, or quality measures. Since not all of those parameters were normally distributed, we also ran add-on Mann-Whitney-U tests which further confirmed that there were no significant group differences (all $p \geq .067$).

**Metabolite**	**Levene's test**	**df**	**One-sided p**	**Two-sided p**
ACC GABA+	Equal variances assumed	62	.270	.540
ACC Glx	Equal variances assumed	62	.104	.207
ACC tNAA	Equal variances assumed	62	.100	.199
SMA GABA+	Equal variances assumed	59	.482	.964
SMA Glx	Equal variances assumed	59	.463	.926
SMA tNAA	Equal variances assumed	59	.487	.973
Striatum GABA+	Equal variances assumed	61	.347	.694
Striatum Glx	Equal variances assumed	62	.270	.540
Striatum tNAA	Equal variances assumed	62	.104	.207
ACC CSF	Equal variances assumed	62	.308	.616
ACC grey matter	Equal variances assumed	62	.446	.892
ACC white matter	Equal variances assumed	62	.308	.615
SMA CSF	Unequal variances assumed	41.7	.159	.318
SMA grey matter	Equal variances assumed	59	.258	.515
SMA white matter	Equal variances assumed	59	.035	.071
Striatum CSF	Equal variances assumed	62	.380	.760
Striatum grey matter	Equal variances assumed	62	.313	.626
Striatum white matter	Equal variances assumed	62	.301	.602
ACC FWHM	Equal variances assumed	62	.290	.580
SMA FWHM	Equal variances assumed	58	.197	.393
Striatum FWHM	Equal variances assumed	62	.209	.417
ACC SNR	Equal variances assumed	62	.045	.091
SMA SNR	Equal variances assumed	58	.277	.554
Striatum SNR	Equal variances assumed	62	.457	.913

References

1. Koyun AH, Stock A-K, Beste C. Neurophysiological mechanisms underlying the differential effect of reward prospect on response selection and inhibition. Sci Rep. 2023;13:10903.
2. Chmielewski WX, Beste C. Testing interactive effects of automatic and conflict control processes during response inhibition - A system neurophysiological study. Neuroimage. 2017;146:1149–1156.
